# Structural Modification of Resveratrol: Biological Activities and Anticancer Potential of Emerging Methoxylated Analogs

**DOI:** 10.3390/ijms27188020

**Published:** 2026-09-09

**Authors:** Yashvi Sethia, Edyta Kopera, Jacek Tabarkiewicz

**Affiliations:** 1 Students’ Scientific Club at Department of Human Immunology, Faculty of Medicine, College for Medical Sciences, University of Rzeszów, 35-959 Rzeszów, Poland; 2 Department of Human Immunology, Faculty of Medicine, College for Medical Sciences, University of Rzeszów, 35-959 Rzeszów, Poland; ekopera@ur.edu.pl

**Keywords:** anticancer, resveratrol derivative, 4,4′-(ethane-1,2-diyl)bis(2-methoxyphenol), 3,5,4′-trimethoxystilbene, antioxidant, plant-derived, bioactive

## Abstract

Resveratrol remains one of the most extensively studied plant-derived polyphenols because of its broad antioxidant, anti-inflammatory, cardioprotective, neuroprotective, and anticancer activities. However, its clinical translation has been substantially limited by its poor aqueous solubility, rapid phase II metabolism, and low systemic bioavailability, despite decades of intensive investigation. Structural modification through methoxylation has emerged as a promising strategy to overcome these pharmacokinetic limitations by enhancing metabolic stability, lipophilicity, membrane permeability, and biological activity. While considerable attention has focused on resveratrol and a limited number of established analogs, emerging methoxylated derivatives remain comparatively underexplored. This review highlights the structural characteristics, biological activities, and molecular mechanisms of two such derivatives, 4,4-(ethane-1,2-diyl)bis(2-methoxyphenol) (EG) and (*Z*)-3,5,4′-trimethoxystilbene (Z-TMS), and discusses evidence from structurally similar, better-characterized compounds that may aid in understanding their activities. Despite promising experimental findings, the therapeutic relevance of EG and Z-TMS remains insufficiently characterized because the available evidence is predominantly derived from in vitro studies, while data on toxicity, therapeutic index, long-term safety, pharmacodynamics, and clinically relevant systemic exposure remain limited. This review emphasizes the anticancer potential of these emerging methoxylated derivatives, identifies current knowledge gaps, and provides a basis for considering them as lead compounds for anticancer drug development.

## 1. Introduction

Resveratrol (3,4′,5-trihydroxy-*trans*-stilbene, RSV) is a widely studied natural polyphenol with considerable therapeutic potential in cancer prevention and treatment [1]. This natural phytoalexin is produced in plant tissues as an evolutionary defense mechanism against environmental stress and microbial attack [1,2,3]. Despite its extensive preclinical success as an anticancer agent, the clinical translation of resveratrol is severely hindered by its poor aqueous solubility (~30 mg/L) [4,5] and rapid phase II metabolism, which result in negligible systemic bioavailability [4,5,6,7,8]. To overcome these pharmacokinetic limitations, structural modifications, particularly methoxylation, have been explored to reduce the susceptibility of hydroxyl groups to metabolic conjugation, thereby enhancing lipophilicity and membrane permeability [9,10,11,12]. This systematic review evaluates the biological activities, synthetic profiles, and therapeutic trajectories of two emerging methoxylated derivatives with distinct geometries: 4,4′-(ethane-1,2-diyl)bis(2-methoxyphenol) (divanillyl; ethylenediguaiacol; EG), a flexible dibenzyl dimer that targets the Akt/mTOR survival pathway [13], and (*Z*)-3,5,4′-trimethoxystilbene (Z-TMS), a rigid cis-stilbene that acts as a potent antimitotic agent via the tubulin colchicine-binding site [14,15].

By benchmarking these compounds against their well-investigated structural counterparts, 3′,4-dihydroxy-3,5′-dimethoxybibenzyl (gigantol) and (*E*)-3,5,4′-trimethoxystilbene (E-TMS) [16,17], this work establishes a comparative framework to guide future lead optimization and clarify the clinical viability of structurally modified stilbenes in targeted therapeutics.

## 2. Resveratrol: Highlighting Limitations

Resveratrol is a natural hydrophobic polyphenolic compound found at relatively high concentrations (50–100 μg/g) in the skin of red grapes (Vitis vinifera) and at variable concentrations in berries such as blueberries, cranberries, and mulberries [2,18]. It is also found in peanuts and peanut-derived products at concentrations of 0.3–15 μg/g and in red wine at concentrations of 0.1–14.3 mg/L [3,4].

Resveratrol has been studied extensively for its potential to delay or prevent the progression of several diseases, including cancer, cardiovascular diseases, and ischemic injury [19,20]. Its effects involve multiple mechanisms, including induction of apoptosis, regulation of the cell cycle, inhibition of inflammation, limitation of angiogenesis, prevention of metastasis, modulation of gene expression, targeting of cancer stem cells, influence on the tumor microenvironment, enhancement of chemotherapeutic efficacy, and modulation of redox and hormone signaling [19,20].

Despite its multifaceted benefits and several preclinical studies on the anticancer activity of resveratrol, the clinical translation of resveratrol remains limited due to several pharmacokinetic drawbacks. One of the main challenges is its very low water solubility (~30 mg/L), which significantly hinders its dissolution and gastrointestinal absorption [5].

Across the current literature, resveratrol has been administered almost exclusively via the oral route in human studies, with no established intravenous (IV) formulations progressing into clinical trials [6]. Although IV administration has been explored in preclinical murine pharmacokinetic studies, these studies have demonstrated only transient increases in parent compound exposure, with rapid clearance and continued conversion to glucuronide and sulfate metabolites [21,22,23]. Even under direct systemic administration, sustained therapeutic plasma concentrations are not maintained, and anticancer efficacy remains inconsistent and model-dependent [6,21]. Upon ingestion, the hydroxyl-rich stilbene core of RSV undergoes rapid first-pass biotransformation in the intestines and liver, where uridine diphosphate-glucuronosyltransferases (UGTs) and sulfotransferases (SULTs) convert the free parent compound into highly water-soluble resveratrol-glucuronides and resveratrol-sulfates [4,6,19].

Table 1 summarizes key clinical trials investigating resveratrol across oncology, cardiometabolic, neurodegenerative, and metabolic diseases, with particular emphasis on limitations. Across all included studies, resveratrol was administered via the oral route, either as standard capsules or formulated preparations (e.g., micronized or nutraceutical formulations).

Across the trials reviewed, the most consistent limitation is resveratrol’s unfavorable pharmacokinetic profile, characterized by rapid phase II glucuronidation/sulfation following oral administration, resulting in low systemic exposure, poor bioavailability, and high interindividual variability. This limitation has been repeatedly highlighted in clinical syntheses; for example, Berman et al. concluded that, despite broad biological activity in preclinical models, resveratrol has failed to demonstrate consistent clinical efficacy across disease areas, with observed effects largely confined to surrogate biomarkers rather than strong clinical endpoints [4,38]. In response to these limitations, research efforts have largely followed two parallel strategies: dose escalation in clinical studies, with oral doses reaching up to 5 g/day, and the development of alternative formulations and analogs, including micronized preparations, nanocarrier systems, and structural derivatives such as methoxylated or modified stilbenes designed to improve pharmacokinetic stability and tissue exposure [8,26,29]. However, most of these approaches remain at preclinical or early-phase clinical evaluation [4,25,39]. To evaluate the dose escalation strategy, Boocock et al. administered single increasing doses up to 5 g in healthy subjects, reaching a peak serum level (C_max_) of only 2.40 μM at 1.5 h (T_max_). Notably, this peak remains below the 5.0–100.0 μM inhibitory threshold (IC_50_) typically required for in vitro anticancer activity, a limitation driven primarily by rapid metabolic clearance rather than poor water solubility [40]. In the same study, the apparent plasma half-life of parent resveratrol following oral administration ranged from approximately 2.9 to 8.9 h across the evaluated doses [40]. Therefore, these findings indicate that dose escalation alone is insufficient to overcome the limitations of resveratrol, reinforcing the need to shift development strategies toward structurally modified derivatives.

## 3. Methoxylated Resveratrol Derivatives

### 3.1. Structural Comparison and Implications for Biological Activity

4,4′-(ethane-1,2-diyl)bis(2-methoxyphenol) (CAS Number 18256-53-6, EG) is a symmetrical dimer linked by a flexible aliphatic chain. The two carbon atoms connecting the aromatic rings are joined by a single covalent sp^3^ bond [9,13]. This configuration gives the molecule flexibility, allowing the two aromatic rings to twist, rotate, and conform dynamically within protein kinase binding pockets. Molecular docking analysis of EG has specifically demonstrated high-affinity interactions with the ATP-binding and allosteric sites of Akt [13].

(*Z*)-3,5,4′-trimethoxystilbene (CAS Number 94608-23-8, Z-TMS) fully replaces all three functional hydroxyl groups with hydrophobic methoxy (-OCH_3_) groups [10]. The elimination of hydrogen-bond donors increases the molecule’s lipophilicity and may facilitate more efficient passage across lipid-rich cell membranes, potentially improving the poor cellular uptake and low bioavailability associated with natural resveratrol [10,11]. The 3,5-dimethoxyphenyl arrangement shares structural similarity with the anticancer drug Combretastatin A-4 [14,17]. Z-TMS binds tightly to the colchicine-binding site on β-tubulin. This binding induces microtubule depolymerization, preventing cancer cells from forming mitotic spindles and resulting in G2/M cell-cycle arrest [14,17].

**Figure 1:**
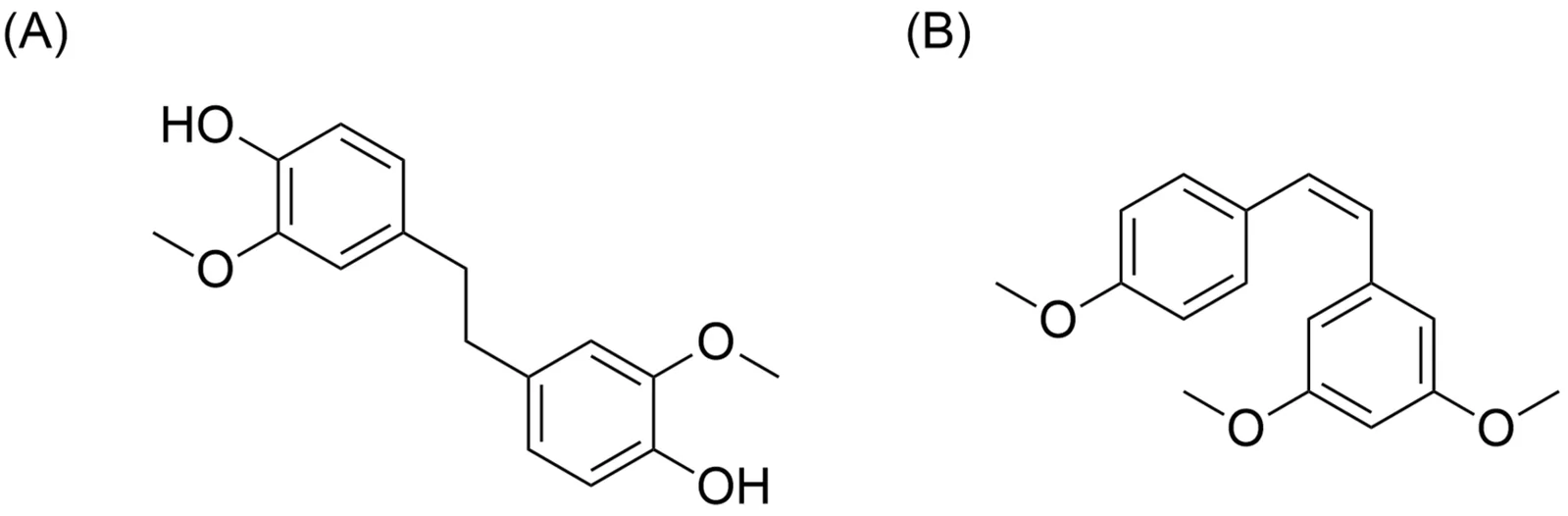
Structures of methoxylated resveratrol derivatives: (**A**) 4,4′-(ethane-1,2-diyl)bis(2-methoxyphenol) (EG); (**B**) (*Z*)-3,5,4′-trimethoxystilbene (Z-TMS).

EG contains a saturated ethane linker that provides rotational flexibility between the aromatic rings, while Z-TMS contains a rigid stilbene double bond that restricts conformational movement and maintains a defined spatial arrangement. Furthermore, the presence of methoxy substitutions and the absence or retention of hydroxyl groups influence hydrogen-bonding potential, lipophilicity, and interaction with molecular targets [9,10]. These structural characteristics provide a basis for understanding the differences in cellular uptake, target affinity, and anticancer mechanisms reported for these derivatives. The two-dimensional structures shown in Figure 1A,B highlight the fundamental chemical differences between EG and Z-TMS.

The distinct structures of EG and Z-TMS result in important differences in physicochemical properties, including molecular flexibility, polarity, hydrogen-bonding capacity, and potential interactions with intracellular targets [9,10,13,41]. Table 2 provides a direct comparison of the structural characteristics of EG and Z-TMS, highlighting how modifications of the parent resveratrol structure may influence biological activity and contribute to differences in their anticancer mechanisms.

The structural divergence between EG and Z-TMS represents two distinct approaches to improving the therapeutic properties of resveratrol. EG retains hydroxyl functionality and incorporates a flexible ethane bridge, which may allow greater conformational adaptability and facilitate accommodation within different molecular binding environments. In contrast, Z-TMS possesses a rigid stilbene backbone and complete methoxylation, resulting in increased lipophilicity, reduced hydrogen-bond donor capacity, and improved membrane permeability compared with hydroxylated resveratrol derivatives [12].

The O-methylation of the stilbene scaffold increases lipophilicity and has been shown to promote cell uptake, protect the compounds from degradation, and improve stability [42,43]. Furthermore, methoxylated derivatives have shown greater bioavailability and total plasma levels of the parent compound in rats [43,44].

These differences may contribute to the distinct biological profiles observed for EG and Z-TMS, with EG potentially favoring modulation of kinase-associated signaling pathways, whereas Z-TMS displays structural characteristics associated with microtubule targeting and mitotic inhibition [13,45].

### 3.2. Sources and Biosynthesis

Innets et al. synthesized EG from the aromatic aldehydes vanillin and isovanillin using a multistep synthetic route. The final step involved catalytic hydrogenation of a stilbene intermediate using 10% palladium on carbon (Pd/C) in tetrahydrofuran (THF) under hydrogen at room temperature for 16 h, producing EG in quantitative yield. The synthesized compound was subsequently characterized by ^1^H and ^13^C NMR, IR spectroscopy, and high-resolution mass spectrometry (HRMS) before being evaluated for its anticancer activity [13].

While traditional production strategies for resveratrol derivatives often rely on engineered microbial fermentation, chemical synthesis using plant biomass offers a highly practical alternative for sourcing 4,4′-(ethane-1,2-diyl)bis(2-methoxyphenol) [41,46,47]. The flexible dimeric bisphenol core of EG can be directly derived from sustainable plant resources and waste biomass. Rather than constructing the molecule biologically, this method uses single-ring bio-aromatics like vanillin and creosol as natural structural platforms. These renewable starting materials naturally contain the required *ortho*-methoxy phenol moieties and can be joined together via a chemical coupling reaction to construct the central saturated ethane bridge [46].

EG can also be accessed via semi-synthetic pathways using abundant, plant-derived platform chemicals [47]. In a study exploring fire-resistant resins, Zavala et al. demonstrated how renewable bio-based hydroxycinnamic acids such as ferulic, p-coumaric, and sinapic serve as valuable precursors for generating specialized methoxyphenol derivatives with customized central bridging groups [47].

Mass-spectrometric data reported in the ACS Energy & Fuels Supporting Information identify EG as a breakdown product formed during the thermal degradation and pyrolysis of woody lignocellulosic biomass [48].

Z-TMS is a naturally occurring methoxylated analog of RSV present in trace amounts in five plant species: *Virola cuspidata*, *Virola elongata*, *Centipeda minima*, *Schoenus nigricans*, and *Rheum undulatum* [49]. However, most available material for biological and chemical studies is obtained synthetically [49,50]. Chabert et al. synthesized Z-TMS via a multistep synthetic route, in which the corresponding *trans*-stilbene was converted to the *cis* isomer through UV-induced photoisomerization. The resulting (*Z*)-3,5,4′-trimethoxystilbene was purified and characterized before evaluation of its antimitotic activity [49].

Kang et al. constructed an artificial biosynthetic pathway in engineered *Escherichia coli* using stilbene synthase and methyltransferase enzymes to produce 3,5,4′-trimethoxystilbene; however, this approach generated the *trans*-(E) isomer rather than the Z-form [41]. Therefore, current approaches for obtaining Z-TMS rely mainly on isolation from natural sources or chemical conversion of the *trans* isomer through photoisomerization [41,49].

### 3.3. Anticancer Activities and Molecular Mechanisms of Action

4,4′-(ethane-1,2-diyl)bis(2-methoxyphenol) (EG) exhibits promising antioxidant and anticancer properties through complementary chemical and cellular mechanisms [13,51]. The antioxidant potential of EG is primarily attributed to its methoxy-substituted bisphenolic structure, which provides two phenolic hydroxyl groups capable of participating in hydrogen atom transfer (HAT)-mediated radical scavenging reactions [51,52]. Through donation of phenolic hydrogen atoms to reactive oxygen species (ROS) and carbon-centered radicals, EG is expected to generate resonance-stabilized phenoxyl radical intermediates, thereby terminating oxidative chain reactions [52]. The presence of two phenolic antioxidant units within a single molecule is predicted to enhance radical-trapping efficiency compared with corresponding monophenolic analogs. This mechanism is supported by studies on structurally related methoxyphenol dimers, where Fujisawa et al. demonstrated that dimerization of 2-methoxy-4-methylphenol (MMP) significantly increased radical-scavenging efficiency, with bis-methoxyphenol derivatives exhibiting approximately twofold higher stoichiometric radical-trapping capacity than their monomeric counterparts [51]. Furthermore, Amorati et al. showed that bisphenolic antioxidants display enhanced radical-scavenging activity due to efficient phenoxyl radical stabilization, intramolecular interactions, and favorable hydrogen transfer kinetics. These findings provide a mechanistic basis for the antioxidant behavior of EG, although direct radical kinetic measurements specifically for EG remain limited [52].

Additionally, EG has demonstrated direct anticancer activity through modulation of the PI3K/Akt/mTOR signaling pathway [13]. Innets et al. investigated EG in human non-small-cell lung cancer (NSCLC) H23 and A549 cells treated with 0–100 μM for 24 h and reported significant inhibition of cancer cell proliferation accompanied by induction of apoptosis. EG reduced phosphorylation of Akt and mTOR, indicating suppression of the Akt/mTOR survival pathway, which plays a central role in regulating cell growth, metabolism, protein synthesis, and resistance to apoptosis [13,53]. For comparison, the cells were also treated with resveratrol. Resveratrol significantly reduced the p-mTOR/mTOR and p-Akt/Akt protein expression ratios at 100 μM, whereas EG significantly reduced the p-Akt/Akt ratio at lower concentrations (50–100 μM) [13].

Molecular docking analysis further suggested that EG may interact with Akt through binding at ATP-associated and allosteric regulatory regions, providing a possible molecular explanation for Akt inhibition. Suppression of Akt activity results in downstream inhibition of mTOR signaling, leading to reduced proliferative signaling and enhanced apoptotic responses in tumor cells [13]. Therefore, EG exhibits a dual mode of biological action: (i) antioxidant activity through phenolic radical scavenging and inhibition of oxidative chain reactions, and (ii) anticancer activity through inhibition of Akt/mTOR-mediated survival signaling. Together, these mechanisms highlight EG as a promising bioactive bisphenolic scaffold with potential applications in oxidative stress-related disorders and cancer therapeutics [13,51,52].

Z-TMS has demonstrated potent anticancer activity, with its pharmacological effects primarily associated with antimitotic activity and disruption of microtubule dynamics [49]. Schneider et al. reported that Z-TMS exhibited markedly enhanced antiproliferative activity compared with resveratrol, producing strong growth inhibition in human colon cancer Caco-2 cells. The increased potency was attributed to structural modification of the stilbene scaffold, particularly methoxylation of the hydroxyl groups and the (*Z*) configuration, which improved biological activity compared with the parent compound [15,49]. Z-TMS demonstrated potent antiproliferative effects, with treatment at 0.3 µM resulting in approximately 80% inhibition of cell growth, while complete growth arrest was achieved at 0.4 µM. At 0.3 µM Z-TMS, cells accumulated at the G2/M transition, demonstrating that the compound interfered with mitotic progression rather than simply causing nonspecific toxicity [15].

Furthermore, Z-TMS inhibited tubulin polymerization in a concentration-dependent manner, with an IC_50_ of approximately 4 µM. Disruption of microtubule assembly prevents proper mitotic spindle formation, leading to failure of cell division and G2/M arrest [15,49]. The antimitotic mechanism of Z-TMS was additionally validated in a study by Mazué et al., which investigated the cell-cycle effects of Z-TMS and related resveratrol analogs in SW480 colorectal cancer cells. Using flow cytometric analysis, they showed that Z-TMS induced accumulation of cells in the M phase and promoted the formation of polyploid cell populations, indicating that cells underwent DNA replication but failed to complete mitosis [54].

Chabert et al. further investigated the antimitotic activity of this resveratrol analog using Caco-2 human colon cancer cells and demonstrated that Z-TMS retained potent antiproliferative activity and was associated with inhibition of mitotic progression. Their study reinforced the importance of structural features, particularly the (*Z*) configuration and trimethoxy substitution pattern, thereby supporting the role of Z-TMS as a microtubule-targeting antimitotic compound [49,55]. Uncontrolled cell-cycle progression is a hallmark of cancer and is regulated by cyclin-dependent kinases (CDKs) and their associated checkpoint proteins [56]. Cyclin-dependent kinase 1 (CDK1) is a key regulator of the G2/M transition and is essential for entry into mitosis, whereas the cyclin-dependent kinase inhibitor p21 (CDKN1A) suppresses CDK activity and promotes cell-cycle arrest [57]. Dysregulated activation of the phosphoinositide 3-kinase (PI3K)/Akt signaling pathway further enhances tumor cell survival and proliferation by promoting cell-cycle progression and inhibiting apoptosis [56,57]. Nguyen et al. investigated hepatocellular carcinoma GS5 and Huh 7.5 cells, as well as the hepatoma cell lines (Huh7 and FCA4) treated with 1 μmol/L Z-TMS for 48 h. The results clearly showed that more than 90% of cells accumulated in the G_2_/M phase, with concomitant reductions in the G_1_ and S phases [45]. In hepatocellular carcinoma cells, Z-TMS reduced CDK1 expression, increased p21 expression, and inhibited Akt phosphorylation, leading to G2/M cell-cycle arrest and suppression of tumor cell proliferation, demonstrating that, beyond disrupting microtubule dynamics, Z-TMS also modulates key molecular regulators governing cell-cycle progression [45].

Cancer stem cells (CSCs) contribute to tumor initiation, progression, metastasis, and therapeutic resistance [56]. Microtubules are highly dynamic cytoskeletal structures that regulate essential cellular processes, including mitotic progression, cell migration and intracellular organization [45]. Doublecortin-like kinase 1 (DCLK1) has emerged as an important cancer stem cell-associated marker in gastrointestinal and hepatocellular cancers and is associated with activation of inflammatory and protumorigenic signals as well as poor prognosis and increased tumor aggressiveness [45,58,59]. The cancer stem cell marker DCLK1 is associated with microtubules and stimulates tubulin polymerization [45]. Nguyen et al. investigated the effects of Z-TMS in DCLK1-overexpressing hepatoma models to determine whether the compound could target cancer stem cell-associated signaling. Treatment with 1 μM Z-TMS disrupted normal DCLK1–microtubule interactions by inducing redistribution of DCLK1 into bundled microtubule structures, accompanied by inhibition of cell proliferation and migration, suggesting that Z-TMS may suppress tumor-promoting characteristics of DCLK1-positive cells through modulation of microtubule dynamics [45].

Colchicine is a microtubule-disrupting compound that binds to β-tubulin and prevents tubulin assembly [15,60]. In addition to its direct effects on microtubule dynamics, competitive binding experiments demonstrated that Z-TMS partially inhibited colchicine binding to tubulin, suggesting that the compound interacts with, or induces conformational changes within, the colchicine-binding region of tubulin [15]. This interaction was also investigated in the study by Mazué et al. where molecular docking studies were performed to compare resveratrol analogs with the colchicine-binding site of tubulin. Their analysis suggested that Z-TMS adopts a favorable orientation within this binding region, with a conformation similar to that of the known antimitotic agent combretastatin A-4 [14,54].

Polyamines, including putrescine, spermidine and spermine, are small polycationic molecules required for DNA replication, RNA transcription, protein synthesis and cell-cycle progression [61,62]. Their intracellular concentrations are tightly regulated by the rate-limiting biosynthetic enzymes ornithine decarboxylase (ODC), which catalyzes the conversion of ornithine to putrescine, and S-adenosylmethionine decarboxylase (SAMDC) [61,63]. Elevated activities of ODC and SAMDC are frequently observed in rapidly proliferating tumor cells, making polyamine biosynthesis an attractive target for anticancer therapy [62,63]. Schneider et al. demonstrated that treatment of Caco-2 cells with 0.3 µM Z-TMS reduced both ODC and SAMDC activities by approximately twofold, resulting in depletion of the intracellular polyamines putrescine and spermidine. These findings suggest that, in addition to disrupting mitosis through inhibition of tubulin polymerization, Z-TMS suppresses tumor cell proliferation by limiting polyamine biosynthesis, thereby reducing the availability of metabolites essential for sustained cancer cell growth [15,63].

Out of the 13 newly synthesized resveratrol analogs, together with known stilbene derivatives, Z-TMS remained the most potent inhibitor of cancer cell proliferation [54].

Beyond its direct anticancer effects, Z-TMS has also demonstrated antiviral activity in the context of hepatitis C virus (HCV)-associated liver disease [45]. Chronic HCV infection promotes persistent hepatic inflammation and is a major risk factor for hepatocellular carcinoma; therefore, compounds capable of simultaneously suppressing viral replication and tumor-associated pathways may have therapeutic value [45,64]. Nguyen et al. tested HCV-replicating hepatoma models, including GS5 and FCA4 cells expressing HCV-1b subgenomic replicons and Huh7.5 cells infected with infectious HCV (JFH1 strain). Z-TMS treatment for 48 h resulted in a concentration-dependent (0.05–2.5 μmol/L) reduction in the HCV NS5B polymerase, an essential viral RNA-dependent RNA polymerase required for genome replication, and decreased HCV RNA levels in infected cells, suggesting that Z-TMS is approximately 100-fold more potent in downregulating the levels of HCV NS5B polymerase [45]. Furthermore, Huh7.5 cells were infected with tissue culture–generated JFH1 viruses (HCVcc) for 48 h to allow replication of the viral RNA. Treatment of infected cells with 1 μmol/L Z-TMS markedly reduced the JFH1 RNA copy number by approximately tenfold [45].

These findings indicate that Z-TMS exhibits both antiviral and antitumor activities, targeting HCV replication while modulating cellular pathways associated with hepatocarcinogenesis.

## 4. Comparative Structural Insights and Mechanistic Relevance of Related Analogs: Rationale for Further Investigation of the Methoxylated Derivatives

**Figure 2:**
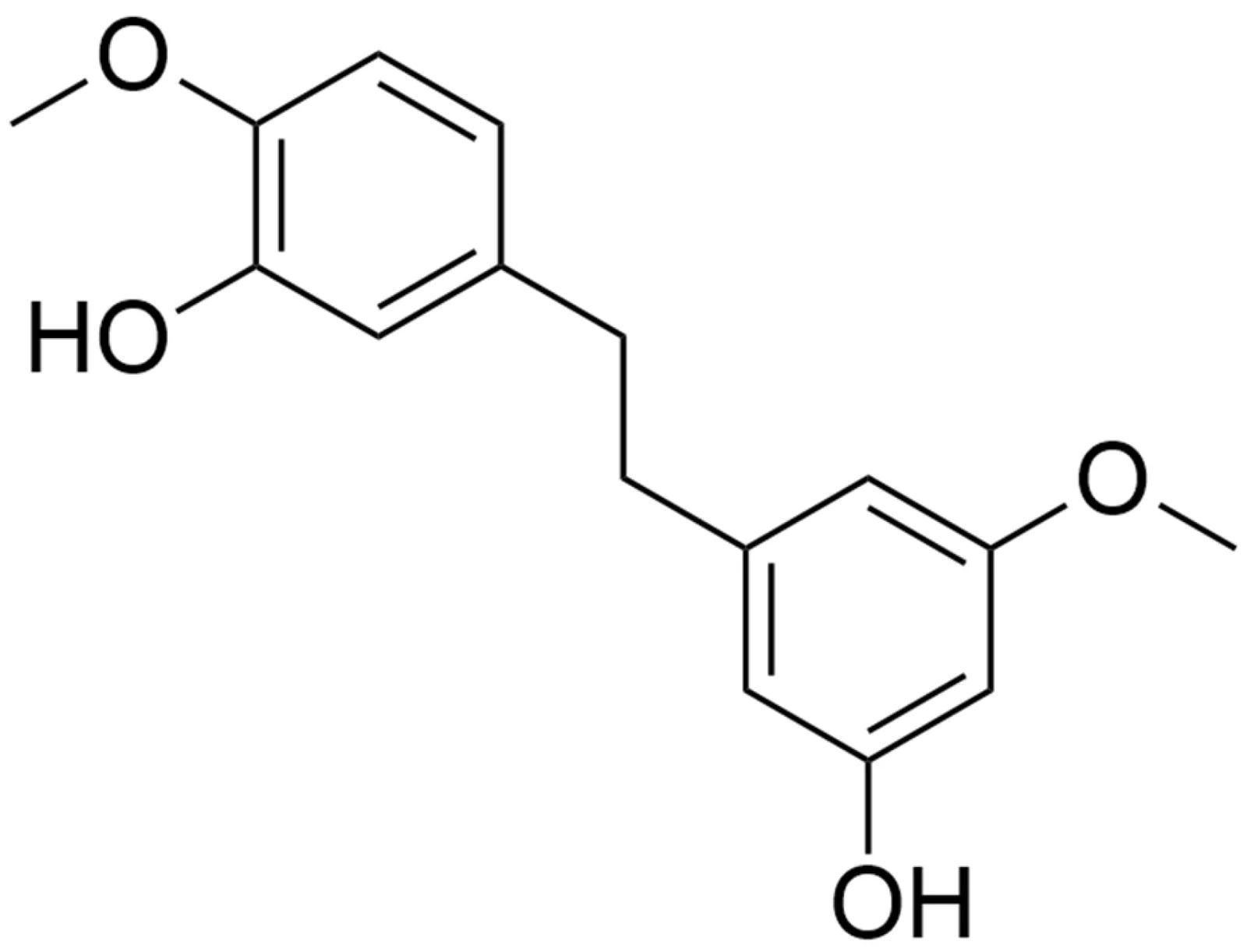
Structure of 3′,4-dihydroxy-3,5′-dimethoxybibenzyl (gigantol).

EG is a bibenzyl derivative structurally related to gigantol (Figure 2). Both compounds share a common bibenzyl scaffold and differ primarily in the relative positioning of the hydroxyl and methoxy substituents, representing closely related positional isomers [9,16]. Gigantol is a naturally occurring bibenzyl compound primarily isolated from orchidaceous plants, particularly those of the *Dendrobium* genus [65].

Preclinical studies indicate that gigantol exhibits broad-spectrum anticancer activity across a range of solid tumor models, including lung, hepatocellular, cervical, breast, and bladder cancers [66,67,68,69,70,71,72]. Although effective concentrations vary depending on the cell type and experimental design, most studies report concentration-dependent inhibition of cancer cell proliferation, migration, invasion, and colony formation, together with induction of apoptosis. These effects are mediated through modulation of several oncogenic signaling pathways, most notably PI3K/Akt/mTOR and Wnt/β-catenin, as well as suppression of epithelial–mesenchymal transition, cancer stem cell properties, and MYC stability [66,67,68,69,70,71,72]. In several models, gigantol demonstrated limited cytotoxicity at lower concentrations while retaining anti-metastatic activity, and in breast cancer cells it enhanced the efficacy of cisplatin, suggesting potential as a chemosensitizing agent [71]. The principal in vitro and in vivo studies investigating the anticancer effects and underlying molecular mechanisms of gigantol are summarized in Table 3.

Collectively, the findings presented in Table 3 highlight gigantol as a promising and extensively researched bibenzyl compound with demonstrated anticancer effects across diverse cell lines [66,67,68,69,70,71,72]. The ability of gigantol to influence multiple cancer-associated processes, including proliferation, apoptosis, metastasis, epithelial–mesenchymal transition, and survival signaling pathways, provides a strong foundation for investigating structurally related compounds [73]. As EG shares a similar framework with gigantol, these findings provide a rationale for exploring EG as a potential bioactive compound. However, the biological effects, molecular targets, and therapeutic potential of EG remain insufficiently characterized [13]. Therefore, the existing evidence for gigantol may serve as a reference framework to guide future studies aimed at determining whether EG can reproduce or improve upon these effects and to define its specific molecular targets and therapeutic potential.

Geometric isomerism represents an important structural determinant in the biological activity of stilbene-based anticancer agents [74]. Z-TMS and its corresponding *E*/*trans* isomer possess an identical chemical composition and substitution pattern, differing in the spatial orientation of the aromatic rings across the central ethylene bridge [10,41]. Compared with Z-TMS, the *trans* isomer (E-TMS) exhibits increased separation between aromatic rings and reduced steric interaction, resulting in a more linear molecular arrangement.

**Figure 3:**
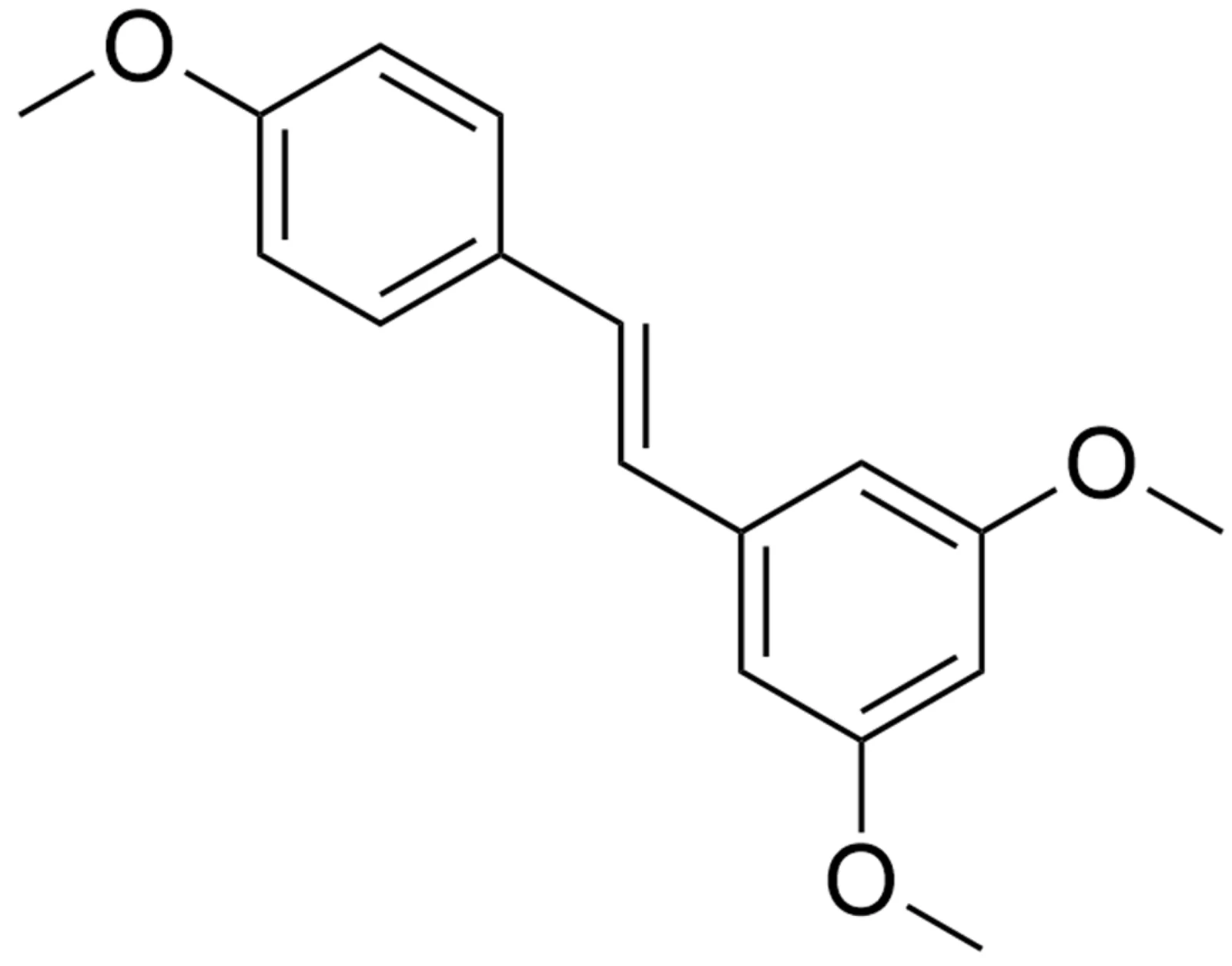
Structure of (E)-3,5,4′-trimethoxystilbene (E-TMS).

E-TMS (Figure 3) exerts potent anticancer activity across multiple tumor types, including non-small cell lung, breast, colorectal, prostate and brain cancers [17,75,76,77,78,79]. Several studies consistently report inhibition of migration, invasion and metastatic potential at relatively low, minimally cytotoxic concentrations, indicating that these effects result primarily from modulation of oncogenic signaling rather than direct cell killing [17,75,76,77,78,79]. The principal molecular targets include the PI3K/Akt, MAPK, NF-κB and Wnt/β-catenin pathways, leading to suppression of epithelial–mesenchymal transition, matrix metalloproteinase activity and other processes associated with tumor progression. At higher concentrations, E-TMS additionally inhibits cell proliferation through induction of cell-cycle arrest and mitochondrial apoptosis [77]. More recent investigations have further expanded these mechanisms by demonstrating regulation of m6A-dependent RNA signaling, modulation of the tumor microenvironment through macrophage reprogramming, and restoration of sensitivity to gefitinib in resistant non-small cell lung cancer models [75,78]. The principal preclinical studies evaluating the anticancer activity and molecular mechanisms of E-TMS are summarized in Table 4.

Across these studies, the available evidence establishes *trans*-3,5,4′-trimethoxystilbene as a well-investigated stilbene derivative with demonstrated anticancer potential across a range of experimental models. In vitro and in vivo studies have shown that E-TMS can inhibit tumor cell proliferation, induce apoptosis, suppress invasion and migration, overcome drug resistance, and interfere with tumor-supportive processes such as angiogenesis and the tumor microenvironment [75,76,77,78,79,80].

However, despite the extensive characterization of the *trans* (*E*) isomer, the corresponding *cis* (*Z*) isomer remains comparatively underexplored. This is particularly noteworthy because the antineoplastic activity of Z-TMS has been reported to exceed that of E-TMS in selected experimental models and to be approximately 100-fold greater than that of resveratrol [50]. Cardile et al. reported greater antiproliferative activity of Z-TMS than E-TMS. The *E*-isomer was converted to the corresponding *Z*-isomer by photoisomerization, and both compounds were evaluated using an MTT-based antiproliferative assay following 72 h of exposure in four human cancer cell lines: DU-145 androgen-independent prostate cancer cells, LNCaP androgen-responsive prostate cancer cells, M-14 melanoma cells, and KB epidermoid carcinoma cells [74]. The compounds were initially tested at 50, 25, 12.5, and 6.25 μM, with (*Z*)-3,5,4′-trimethoxystilbene additionally evaluated at lower concentrations (0.5, 0.1, 0.05, and 0.025 μM) due to its higher potency. The *Z*-isomer demonstrated greater antiproliferative activity than the *E*-isomer in DU-145, LNCaP, and particularly KB cells [74]. Further confirmation of the influence of stilbene geometry on anticancer activity was provided by Mazué et al. when 14 resveratrol analogs were analyzed, including the *E*- and *Z*-isomers of 3,5,4′-trimethoxystilbene, in SW480 colorectal cancer cells. Consistent with the findings of Cardile et al., the (*Z*)-3,5,4′-trimethoxystilbene isomer demonstrated superior antiproliferative activity compared with its *E* counterpart.

These comparative studies, which suggest enhanced potency of the *cis* isomer in selected experimental models, highlight Z-TMS as a promising candidate for further evaluation. Thus, the established anticancer profile of *trans*-3,5,4′-trimethoxystilbene provides a rationale for expanding research towards Z-TMS to determine whether the *Z* configuration may confer additional therapeutic advantages.

Importantly, the biological activities and mechanisms reported for structurally related compounds are discussed here as comparative evidence and as a basis for generating hypotheses regarding EG and Z-TMS; they should not be interpreted as direct experimental evidence for either methoxylated derivative. The biological effects of EG and Z-TMS must be established independently through compound-specific experimental studies.

## 5. Anticancer Potential and Future Directions

EG has emerged as one of the more promising resveratrol derivatives for anticancer activity, owing to its mechanism-based inhibition of oncogenic signaling pathways and its potential as a lead for targeted therapeutic optimization [13]. The anticancer activity of naturally occurring polyphenols is commonly associated with their antioxidant capacity and pleiotropic modulation of cellular signaling pathways; however, EG has been distinguished as a synthetic derivative exhibiting a more mechanism-oriented pharmacological profile through selective interference with Akt/mTOR signaling [13,81,82]. The identification of Akt as a molecular target is particularly noteworthy given the central role of Akt/mTOR signaling in tumor cell survival, proliferation, therapeutic resistance, and disease progression across multiple cancer types, suggesting that EG may have broader applicability beyond the non-small cell lung cancer models in which it has been evaluated [13].

To date, the characterization of EG is based on a single comprehensive in vitro study [13], highlighting the need for further independent in vitro investigations to confirm and validate its reported biological effects and to assess the reproducibility of its activity across different experimental models. In addition, expanded in vitro evaluation using diverse cancer and noncancerous cell systems would be valuable to determine whether its antioxidant and signaling-related properties are consistently retained and translatable across biological contexts. Importantly, the current lack of in vivo evidence necessitates further preclinical studies to investigate its pharmacokinetic profile, including oral bioavailability, metabolic stability, and systemic exposure, which are critical parameters for assessing its suitability for therapeutic development. The absence of in vivo efficacy data is accompanied by a largely unexplored safety and toxicological profile. Evaluation of EG in metastasis-related experimental models, including cell migration and invasion assays, may also provide additional insight into its anticancer potential.

Although EG remains at an early stage of development, its mechanism-based activity and favorable structural characteristics identify it as a promising starting point for the development of Akt-targeted anticancer agents [13].

Z-TMS is emerging as a unique therapeutic candidate among methoxylated resveratrol analogs, demonstrating potent antiproliferative and antimitotic activity at submicromolar concentrations across multiple cancer models while targeting fundamental cellular processes [15,45,49].

Despite the remarkable preclinical anticancer activity of resveratrol, its clinical translation has remained limited. Even with IV administration, resveratrol fails to achieve sustained therapeutic plasma concentrations [6,19]. In this context, Lin et al. developed a simple and sensitive high-performance liquid chromatography (HPLC) method for the determination of Z-TMS in rat plasma and applied it to a preclinical pharmacokinetic evaluation. The assay demonstrated excellent analytical performance, exhibiting analytical recoveries (94.6 ± 9.1% to 97.0 ± 2.1%), confirming that Z-TMS could be quantified accurately and reproducibly in biological samples [50]. Subsequently, Z-TMS was administered to rats via both oral and IV routes. While oral administration resulted in negligible systemic bioavailability, IV dosing generated quantifiable plasma concentration-time profiles suitable for pharmacokinetic analysis. The IV profile demonstrated measurable systemic exposure with a prolonged terminal elimination phase, as reflected by extended half-life values derived from the terminal slope of the concentration–time curve, along with comparatively reduced clearance [50].

Therefore, although Z-TMS demonstrated a relatively favorable pharmacokinetic profile following intravenous administration, including a prolonged terminal elimination half-life and measurable systemic exposure, its negligible oral bioavailability represents an important limitation for its potential therapeutic development by the oral route. These findings highlight the importance of considering the route of administration and formulation when evaluating the pharmacological potential of Z-TMS. The study by Lin et al. provided an important pharmacokinetic foundation for subsequent efficacy, toxicological, and dose-optimization studies; however, further investigations are required to establish whether adequate systemic exposure can be achieved through clinically relevant routes of administration and to determine the relationship between pharmacokinetic exposure, efficacy, toxicity, and therapeutic index.

Epidermal growth factor receptor (EGFR) is a transmembrane tyrosine kinase that plays a central role in regulating cellular proliferation, survival, and differentiation [83]. Activating mutations or overexpression of EGFR are frequently observed in a range of malignancies and are strongly associated with uncontrolled tumor growth, therapeutic resistance, and poor clinical prognosis [83]. Nguyen et al. used the well-characterized erlotinib-resistant NSCLC cell line H1975, which harbors the T790M gatekeeper mutation in EGFR, to determine the effect of Z-TMS on these cells. The results indicated that Z-TMS significantly reduced survival of H1975 cells in the 0.1 μM to 1.0 μM range within 48 h, whereas erlotinib failed to reduce cell survival even though it was fully active against the wild-type EGFR kinase domain [45]. This finding is potentially relevant to drug-resistant NSCLC because Z-TMS retained cytotoxic activity in EGFR T790M-mutant, erlotinib-resistant H1975 cells, supporting further investigation of its activity in drug-resistant tumor models [45].

Despite these compelling findings, the therapeutic potential of Z-TMS remains supported by relatively limited evidence. Future investigations should therefore focus on validating its efficacy across a broader range of cancer models, while comprehensively characterizing its therapeutic index, long-term safety, and pharmacodynamic profile. In vitro and in vivo studies are warranted to confirm its effects on apoptosis, cell cycle regulation, and other downstream signaling pathways, thereby facilitating its progression toward clinical evaluation. Furthermore, evaluating the microtubule-disrupting property of Z-TMS in combination with established chemotherapeutic agents or targeted therapies may identify synergistic interactions capable of improving therapeutic efficacy while potentially reducing the doses required for conventional treatments.

Although both EG and Z-TMS have been investigated as structural modifications intended to improve the pharmacological limitations of resveratrol, available evidence suggests that differences in linker structure and conformational flexibility may influence their biological activity and pharmacokinetic profiles [13]. Z-TMS currently benefits from a comparatively more substantiated evidence base linking its systemic exposure to functional anticancer activity [15,45,49,50,54]. In contrast, EG remains less extensively characterized in in vitro and in vivo studies using clinically relevant cancer models, making its translational trajectory less clearly defined at present [13]. Nevertheless, direct comparative in vitro and in vivo studies assessing antiproliferative efficacy, cell cycle modulation, and underlying molecular mechanisms between these analogs are still required to accurately delineate their relative clinical potential and to guide future lead optimization within resveratrol-derived analogs.

For both EG and Z-TMS, the relationship between their observed in vitro activities and achievable in vivo exposure remains insufficiently established, while their selectivity toward cancer versus noncancerous cells and their safety profiles require further investigation.

Although the available literature on the two methoxylated derivatives is predominantly focused on their anticancer properties, limited evidence suggests that their biological activities may extend beyond oncology. In particular, the reported antiviral activity of Z-TMS indicates the potential for non-oncological applications. However, the current evidence remains insufficient to draw broader conclusions regarding other therapeutic applications. Further experimental studies are therefore warranted to systematically investigate the non-oncological biological activities of these derivatives and to determine whether their pharmacological potential extends beyond anticancer applications.

## 6. Conclusions

The available evidence supports EG and Z-TMS as structurally distinct methoxylated derivatives with anticancer activities warranting further investigation. For EG, compound-specific evidence remains limited and is based predominantly on findings from a single in vitro study, including reported modulation of Akt/mTOR-associated signaling. Z-TMS has been evaluated across a broader range of experimental models, with demonstrated antiproliferative and antimitotic activity. Nevertheless, its negligible oral bioavailability and the limited evidence regarding long-term safety and therapeutic index remain important barriers to translation.

Evidence derived from the structurally related compounds gigantol and E-TMS provides useful comparative context but should not be interpreted as demonstrating equivalent biological activities or mechanisms for EG and Z-TMS. Accordingly, proposed relationships between structural similarity and additional therapeutic activities remain hypothesis-generating until confirmed through compound-specific studies.

Overall, the current evidence supports continued investigation rather than conclusions regarding clinical efficacy. Independent validation of EG, expanded in vivo evaluation, compound-specific pharmacokinetic and toxicological characterization, and further assessment of Z-TMS efficacy and safety are required to determine whether the promising experimental properties of these derivatives can translate into therapeutically relevant advantages.

## 7. Materials and Methods

### 7.1. Review Design

This systematic review was conducted using a structured literature search and evidence synthesis approach and was reported with reference to the Preferred Reporting Items for Systematic Reviews and Meta-Analyses (PRISMA) 2020 statement [84], with disclosed limitations. The review aimed to identify and synthesize evidence concerning the pharmacokinetic and biological limitations of resveratrol and the structural, pharmacological, and therapeutic properties of selected methoxylated resveratrol derivatives, 4,4′-(ethane-1,2-diyl)bis(2-methoxyphenol) (EG) and (*Z*)-3,5,4′-trimethoxystilbene (Z-TMS), together with relevant structurally related compounds. A formal review protocol was not prospectively registered.

### 7.2. Information Sources and Search Strategy

Literature searching was undertaken between 1 June and 30 July 2026. PubMed/MEDLINE and Scopus were used as the principal bibliographic sources. Electronic database searches were supplemented by web-based searches and manual screening of the reference lists of relevant primary studies and review articles to identify additional potentially relevant publications.

The searches were conducted as a series of targeted keyword searches reflecting the principal compounds and concepts addressed in the review. The major search terms and combinations included “resveratrol limitations,” “resveratrol derivatives,” “3,5,4′-trimethoxystilbene,” “Z-3,5,4′-trimethoxystilbene,” “TMS AND resveratrol,” “gigantol,” “gigantol AND resveratrol,” and “4,4′-(ethane-1,2-diyl)bis(2-methoxyphenol).” Relevant spelling and naming variants encountered during the search were additionally considered.

The search strategy was intentionally broad because the scope of the review encompassed pharmacokinetic limitations of resveratrol, structural modification, methoxylated derivatives, biological and pharmacological activity, anticancer effects, and molecular mechanisms. Reference lists of relevant publications were additionally screened to identify studies not retrieved through the principal keyword searches.

The original electronic search histories were not retained; consequently, exact database-specific retrieval counts could not be retrospectively verified, and a formal study-selection flow diagram could not be reconstructed.

### 7.3. Eligibility Criteria

Original experimental or clinical studies were considered eligible when they provided evidence directly relevant to the objectives of the review. Eligible studies included those addressing one or more of the following: pharmacokinetic or bioavailability limitations of resveratrol; structural modification of resveratrol; methoxylated resveratrol derivatives or relevant structural analogs; biological or pharmacological activity; anticancer effects; molecular mechanisms of action; or therapeutic potential.

Clinical studies and experimental in vivo and in vitro studies were eligible when sufficient information was available regarding the investigated compound, experimental or clinical model, intervention or exposure, and relevant structural, pharmacokinetic, biological, pharmacological, or mechanistic outcomes.

Studies were excluded when they did not address the objectives of the review; focused exclusively on physical drug-delivery systems such as formulations, liposomes, or nanoparticles without relevant evaluation of the properties of the compound itself; concerned industrial or other non-biological applications without relevant pharmacological or biological endpoints; or provided insufficient information for meaningful interpretation of the reported findings. Review articles were used for contextual information and reference-list searching but were not treated as primary experimental evidence in the qualitative synthesis.

### 7.4. Study Selection

Study selection was performed by one reviewer (Y.S.). Titles and abstracts of potentially relevant publications were initially evaluated according to the eligibility criteria. Potentially eligible publications were subsequently assessed using the available full text, and final inclusion was determined according to their relevance to the predefined objectives and eligibility criteria.

Because study selection was performed by a single reviewer, no procedure for resolving disagreements between reviewers was applicable. Final decisions regarding inclusion or exclusion were made by Y.S.

### 7.5. Data Extraction

Data extraction was performed by one reviewer (Y.S.). Information was collected according to the objectives of the review and included, where available, the author and publication year; investigated compound or derivative; structural characteristics; experimental or clinical model; disease or cancer type; cell line, animal model, or human population; intervention, dose, or concentration; comparator or control; principal biological or pharmacological outcomes; molecular targets and signaling pathways; proposed mechanisms of action; pharmacokinetic and bioavailability findings; toxicity or adverse effects; and principal findings and limitations.

The extracted information was organized according to the principal compounds and themes addressed in the review and summarized descriptively in the corresponding tables and narrative synthesis.

### 7.6. Quality and Risk-of-Bias Assessment

A formal study-level risk-of-bias assessment using standardized design-specific instruments was not performed. The evidence included in the review was methodologically heterogeneous and encompassed clinical studies, animal experiments, in vitro investigations, and structural and mechanistic studies.

The absence of a formal risk-of-bias assessment is acknowledged as a methodological limitation. Furthermore, because study selection and data extraction were performed by a single reviewer, independent verification of eligibility decisions and extracted information was not performed, and reviewer-related selection or extraction bias cannot be excluded.

### 7.7. Evidence Synthesis

Because of substantial heterogeneity among the included studies with respect to study design, experimental model, investigated compound, dose or concentration, treatment duration, biological endpoints, and molecular mechanisms, quantitative meta-analysis was not considered appropriate. The evidence was therefore synthesized qualitatively.

The synthesis was organized according to the major themes of the review, including the pharmacokinetic and bioavailability limitations of resveratrol, structural approaches intended to overcome these limitations, the characteristics of methoxylated resveratrol derivatives, the biological and anticancer activities of EG and Z-TMS, and evidence concerning related resveratrol analogs.

## Figures and Tables

**Table 1:** Clinical trials and human volunteer studies on resveratrol [7,8,24,25,26,27,28,29,30,31,32,33,34,35,36,37].

#	Study	Condition/Population	Dose & Duration	Sample Size	Structural/Solubility/Bioavailability Limitations	Other Limitations
1	Holcombe et al., 2009 [24] (phase I pilot clinical trial, non-randomized) NCT00256334	Colorectal cancer chemoprevention	20 or 80 mg/day; 2 weeks	8 patients	Resveratrol metabolized, mostly into sulfate conjugates and glucuronide; undergoes extensive phase II glucuronidation and sulfation, resulting in low systemic availability [24,25].	Small sample size; possible confounding by diet or drug use
2	Patel et al., 2010 [7] (phase I, non-randomized)	Colorectal cancer	0.5 or 1.0 g/day; 8 days	20 patients	Parent resveratrol in plasma was below the limit of quantitation (LOQ) even at 1.0 g/day; the dominant species in plasma and colorectal tissue was the highly conjugated resveratrol sulfate glucuronide (13.4–22.3 nmol/mL), showing that the hydroxyl-rich stilbene structure undergoes extremely rapid phase II metabolism [7].	Short treatment duration (8 days); uncertainty regarding whether metabolite concentrations achieved a therapeutic effect
3	Howells et al., 2011 [26] (phase I randomized pilot trial) NCT00920803	Colorectal cancer with hepatic metastases	5.0 g/day SRT501; 14 days	6 patients	SRT501 is a micronized oral formulation created specifically because RSV has poor aqueous solubility and rapid metabolism; only marginally increased cleaved caspase-3 in malignant hepatic tissue [26].	Very small pilot sample (*n* = 6); marginal changes in Ki-67 and cleaved caspase-3
4	Timmers et al., 2011 [27] (randomized, placebo-controlled, double-blind crossover study)	Obese men (metabolic effects)	150 mg/day; 30 days	11 subjects	Beneficial metabolic findings hypothesized to be dose-dependent—oral bioavailability shown to be non-linear; effective intracellular concentrations are difficult to achieve with the parent structure [27].	Results not reproduced by Poulsen et al.; limited generalizability
5	Poulsen et al., 2013 [8] (high dose) (randomized, placebo-controlled, double-blind parallel-group trial) NCT01150955	Obese, otherwise healthy men	1.5 g/day high dose; 4 weeks	24 men	At the highest tolerable oral dose, free resveratrol was undetectable; only extensive glucuronide/sulfate metabolites and dihydroresveratrol were measurable [8].	Short duration; null findings for insulin sensitivity and CV risk; conflicting with those of Timmers et al. [27]
6	Kjaer et al., 2015 [28] (phase II randomized, placebo-controlled, double-blind exploratory)	Prostate cancer	150 mg or 1000 mg/day; 4 months	66 patients	High clearance from the circulation and low bioavailability due to phase II metabolism make therapeutic plasma concentrations difficult to sustain and may explain the modest antiandrogen effects without tumor shrinkage [25,28].	No reduction in PSA levels or prostate volume; limited viability as monotherapy
7	Popat et al., 2013 [29] (phase II, open-label, single-arm study) NCT00920556	Relapsed/refractory multiple myeloma (SRT501 ± bortezomib)	5 g/day SRT501; 21 days per cycle, up to 12 cycles	24 patients	SRT501 was developed to improve oral bioavailability; despite this, treatment was associated with substantial gastrointestinal toxicity and renal adverse events, limiting clinical utility [29].	Unacceptable safety profile; ORR of 22% (mITT); nausea in 79% and diarrhea in 71% of patients; potentially excessive dose of 5 g/day
8	Zhu et al., 2012 [30] (phase I/II biomarker study, randomized, placebo-controlled)	Breast cancer	5 or 50 mg twice daily; 12 weeks	39 patients	Even at 100 mg/day, the study did not establish a clear dose-dependent biomarker effect in breast tissue, suggesting that tissue exposure may have been inadequate for consistent biological effects [30].	Small sample size; unclear clinical significance of methylation changes
9	van der Made et al., 2015 [31] (randomized, placebo-controlled, crossover trial) NCT01364961	Overweight/slightly obese subjects	150 mg/day; 4 weeks	45 subjects	Free resveratrol was not detectable except in all plasma samples from one subject. Unabsorbed RSV can be reduced by specific gut microbiota (e.g., *Slackia* and *Adlercreutzia*) to dihydroresveratrol (DHR), contributing to substantial interindividual variability in plasma profiles (0–1080 ng/mL total resveratrol; 0–1070 ng/mL DHR) [31].	Insufficient statistical power; substantial interindividual variability; null cardiovascular findings
10	Tomé-Carneiro et al., 2013 [32] (randomized, placebo-controlled, triple blind trial) Spanish National Research Project BFU2007-60576	Stable coronary artery disease	Grape resveratrol nutraceutical; 1 year	75 patients	The grape matrix was proposed to improve resveratrol bioavailability by co-delivering quercetin and other bioactives that inhibit RSV metabolism [32].	Small sample (n = 75); 1-year follow-up; 16% event rate; lack of statistical adjustment for medications
11	Imamura et al., 2017 [33] (randomized, placebo-controlled, double-blind trial)	Type 2 diabetes (arterial stiffness)	Resveratrol; 12 weeks	T2DM patients	Could not establish whether improvements in arterial stiffness reflected direct vascular exposure to resveratrol [33].	Single-center; arbitrary CAVI cutoff; absence of crossover design
12	Bo et al., 2018 [34] (randomized, placebo-controlled, double-blind trial) NCT01492114	Type 2 diabetes (bone health)	500 mg/day RSV; long-term	T2DM patients	At 500 mg/day, free resveratrol is still poorly absorbed, so the dose reaching bone remodeling sites was uncertain [34].	Greater DXA precision error with elevated BMI; absence of bone turnover markers; subgroup analyses based on small samples
13	Moussa et al., 2017 [35] (phase II randomized, placebo-controlled, double-blind trial) NCT01504854	Mild-to-moderate Alzheimer’s disease	High-dose oral RSV; 52 weeks	104 patients (56 RSV, 48 placebo)	Resveratrol’s poor CNS penetration is partly structural—rapid glucuronidation/sulfation outside the brain limits the fraction that crosses the BBB; altered CSF biomarker trajectories did not translate to clinical cognitive benefit [35,36].	Study designed primarily for safety and tolerability; no effects on MMSE, ADAS-cog, or CDR-SB; weight loss reported as an adverse event; small sample size
14	McCreary et al., 2022 [37] (phase II, randomized, placebo-controlled, double-blind trial) NCT04400890	Mild COVID-19 (outpatient)	Oral RSV; standard course	100 patients (terminated early after futility)	Stated that much of the resveratrol literature is concerned about poor bioavailability and noted that several studies dismiss resveratrol-glucuronide metabolites; molecular docking suggests glucuronides may have higher antiviral binding affinity than parent RSV [37].	Trial terminated early for futility; FDA-imposed cap of 200 patients; limited geographic and racial diversity; possible need for a higher dose in hospitalized patients with obesity because of the larger volume of distribution

Abbreviations: ADAS-cog, Alzheimer’s Disease Assessment Scale–cognitive subscale; BBB, blood–brain barrier; BMI, body mass index; CAVI, cardio-ankle vascular index; CDR-SB, Clinical Dementia Rating–Sum of Boxes; CNS, central nervous system; CV, cardiovascular; DHR, dihydroresveratrol; DXA, dual-energy X-ray absorptiometry; FDA, Food and Drug Administration; LOQ, limit of quantitation; mITT, modified intention-to-treat; MMSE, mini-mental state examination; ORR, objective response rate; PSA, prostate-specific antigen; RSV, resveratrol; T2DM, type 2 diabetes mellitus.

**Table 2:** Structural comparison of EG and Z-TMS.

Structural Feature	EG	Z-TMS
Molecular Formula	C_16_H_18_O_4_	C_17_H_18_O_3_
Molecular Weight	274.31 g/mol	270.32 g/mol
Aliphatic Bridge	Saturated ethane (-CH_2_-CH_2_-) single bond	Unsaturated ethene (-CH=CH-) bond
Conformational Flexibility	High (free rotation around the single bond)	Rigid (fixed *cis* (*Z*) geometry due to restricted rotation)
Ring Symmetry	Symmetrical (two identical)	Asymmetrical (two non-equivalent aromatic rings)
Total Hydroxyl (-OH) Groups	Two (one per ring, at the 4 and 4′ positions)	None (fully methylated molecule)
Total Methoxy (-OCH_3_) Groups	Two (one per ring, at the 2 and 2′ positions)	Three groups total (at the 3, 5, and 4′ positions)

-CH_2_-CH_2_-, saturated ethane bridge; -CH=CH-, unsaturated ethene bridge; -OH, hydroxyl group; -OCH_3_, methoxy group. Molecular weights are expressed as approximate calculated averages based on standard atomic weights.

**Table 3:** Summary of anticancer activities and molecular mechanisms of gigantol in different cancer cell models [66,67,68,69,70,71,72].

#	Study	Cancer Model/Cell Line (s)	Gigantol Concentration(s)	Biological Activity and Mechanism	Main Findings
1	Charoenrungruang et al., 2014 [66]	NSCLC H460	10, 50, 100, and 500 µM	Anti-migratory; suppression of filopodia formation; ↓ Caveolin-1 (Cav-1), ↓ Cdc42 signaling, modulation of Akt signaling [66]	Gigantol significantly inhibited migration of H460 cells without marked cytotoxicity at lower concentrations, indicating an anti-metastatic effect [66].
2	Losuwannarak et al., 2019 [67]	NSCLC H460, nude mouse xenografts	0–200 µM	Cancer stem cell inhibition; ↓ PI3K/Akt/mTOR, ↓ JAK/STAT signaling, ↑ JNK; reduced tumor maintenance [67].	Pretreatment with gigantol suppressed tumor growth, reduced tumor density, and attenuated the tumor maintenance in NSCLC xenografts [67].
3	Losuwannarak et al., 2020 [68]	NSCLC H460, A549, H292	0–200 µM	Antiproliferative; MYC degradation via ubiquitin-proteasome pathway; colony formation inhibition [68].	Gigantol treatment at concentrations lower than 20 μM caused no significant reduction in cell viability, whereas 50–200 μM caused significant toxic effects in a dose-dependent manner. Gigantol inhibited lung cancer proliferation by reducing MYC stabilization through enhancing GSK3β-mediated ubiquitin-proteasomal degradation [68].
4	Chen et al., 2017 [69]	Human hepatocellular carcinoma HepG2	0–150 µM	Suppression of the PI3K/Akt/NF-κB signaling pathway; reduction in Akt phosphorylation; activation of apoptosis-related proteins including caspase-3, PARP, and p53 [69].	Gigantol significantly inhibited HepG2 cell proliferation and induced apoptosis [69].
5	Kang et al., 2022 [70]	Human cervical cancer, HeLa cells	0, 10, 20, 40, 80, and 160 µM	Antiproliferative and pro-apoptotic activity, modulation of Wnt/β-catenin signaling pathway; induction of reactive oxygen species (ROS)-mediated apoptosis; regulation of apoptosis-related proteins [70]	Gigantol inhibited HeLa cell proliferation in a concentration-dependent manner and increased apoptosis, with these effects associated with increased ROS generation, mitochondrial apoptotic signaling, and suppression of Wnt/β-catenin pathway activity [70]. Reduced β-catenin-related signaling, contributing to inhibition of cervical cancer cell growth [70].
6	Huang et al., 2021 [71]	Human breast cancer MCF-7 and MDA-MB-468	Broad range tested in proliferation up to 320 µM. Combined experiments used gigantol with cisplatin (DDP) to examine chemosensitization.	Downregulation of the PI3K/Akt/mTOR signaling pathway; reduced activation/phosphorylation of PI3K, Akt, and mTOR; pathway involvement [71].	Gigantol inhibited breast cancer cell growth and increased the apoptotic effect of DDP. The combination treatment produced stronger anticancer effects than either treatment alone, suggesting gigantol acts as a cisplatin sensitizer by suppressing PI3K/Akt/mTOR survival signaling [71].
7	Zhao et al., 2020 [72]	Human bladder cancer cell lines SW780, 5637, and T24	0, 40, 80, and 160 µM	Inhibition of Wnt signaling and epithelial–mesenchymal transition (EMT). Gigantol reduced expression of Wnt target genes (Axin2, Survivin) and EMT markers (Slug, Vimentin) [72].	Gigantol produced a significant dose- and time-dependent decrease in cell viability. It exhibited lower toxicity toward higher-grade T24 cells than toward lower-grade bladder cancer cells, with an observable reduction in T24 cell growth only at 160 μM; antimetastatic activity, growth inhibition, and enhanced apoptosis were also reported [72].

Abbreviations: ↑, upregulation/increase; ↓, downregulation/decrease; Cav-1, caveolin-1; Cdc42, cell division control protein 42; PI3K, phosphoinositide 3-kinase; Akt, protein kinase B; mTOR, mechanistic target of rapamycin; JAK/STAT, Janus kinase/signal transducer and activator of transcription; JNK, c-Jun N-terminal kinase; MYC, MYC proto-oncogene; GSK3β, glycogen synthase kinase 3 beta; NF-κB, nuclear factor kappa B; PARP, poly(ADP-ribose) polymerase; ROS, reactive oxygen species; EMT, epithelial–mesenchymal transition; Wnt, wingless-related integration site; β-catenin, beta-catenin; DDP, cisplatin. Concentration refers to those reported in the original studies.

**Table 4:** Summary of the anticancer activities and molecular mechanisms of (E)-3,5,4′-trimethoxystilbene (E-TMS) in different cancer cell models [17,75,76,77,78,79].

#	Study	Cancer Model/Cell Line (s)	Concentration(s)	Biological Activity and Mechanism	Main Findings
1	Liu et al., 2024 [75]	NSCLC cell lines H1299, H1975, A549, H460; H1299 xenograft mice model	in vitro: 5 μM (main functional assays) In vivo: 10, 30, and 50 mg/kg	Suppresses NSCLC progression by reducing m6A-modified circPACRGL, inhibiting IGF2BP2-mediated YAP1 mRNA stabilization [75].	E-TMS inhibited NSCLC growth, migration and invasion. In mice, E-TMS reduced tumor volume and tumor weight. The antitumor effect was mediated not only by tumor-cell inhibition but also by reprogramming tumor-associated macrophages toward a less tumor-promoting phenotype [75].
2	Tsai et al., 2013 [76]	Human breast cancer cells MCF-7	0.5–10 μM	Anti-invasive activity, suppresses PI3K/Akt signaling, reduces Wnt/β-catenin pathway activation, reverses EMT markers (reduced mesenchymal phenotype), thereby decreasing migration/invasion [76].	E-TMS inhibited breast cancer invasiveness through modulation of signaling pathways rather than through cytotoxicity alone, highlighting its effects on metastatic behavior [76].
3	Yang et al., 2009 [77]	Human lung adenocarcinoma cells A549	1–100 μM tested for cytotoxicity; 5 μM used for functional and molecular mechanism analyses	Anti-invasive and anti-metastatic, inhibited cell adhesion, migration and invasion by suppressing the JNK/p38 MAPK → NF-κB/AP-1 → MMP-2 signaling pathway, resulting in reduced MMP-2 expression and activity [77].	At 5 μM E-TMS significantly inhibited A549 cell adhesion, migration, and invasion without causing marked cytotoxicity, suggesting that its antimetastatic effect was due to signaling inhibition rather than cell death. Increasing concentrations caused greater inhibition of A549 cell viability, suggesting additional cytotoxic effects at higher doses [77].
4	Lu et al., 2019 [78]	NSCLC cell line H1299 and PC-9/GR (gefitinib-resistant)	0, 0.5, 5, 50, and 500 μM tested for dose–response; 5 μM E-TMS used for functional studies	Anticancer and gefitinib-sensitizing activity; increased apoptosis and reduced resistance to gefitinib by blocking the PI3K/Akt and MAPK pathways [78].	E-TMS enhanced gefitinib sensitivity, inhibited NSCLC cell growth, promoted apoptosis [78].
5	Zielińska-Przyjemska et al., 2017 [17]	Human glioblastoma T98G cells	1, 10, 30, 50, and 100 μM	Antiproliferative and pro-apoptotic activity, induced G2/M cell-cycle arrest, increased apoptotic cell death, promoted mitochondrial membrane potential loss, increased phosphatidylserine externalization, and increased cleaved caspase-3 [17].	E-TMS strongly inhibited proliferation by causing accumulation of cells in the G2/M phase and activating apoptosis-related pathways. The strongest apoptotic response among tested stilbenes was observed with E-TMS and was associated with p53 induction [17].
6	Pan et al., 2008 [79]	Human colorectal cancer COLO 205, HT-29 and prostate cancer PC-3 cells	COLO 205 IC_50_ = 6.25 μM HT-29 IC_50_ = 81.31 μM PC-3 IC_50_ = 42.71 μM	Antiproliferative and pro-apoptotic activity. E-TMS induced ROS generation, mitochondrial dysfunction, cytochrome-c release, caspase activation and DNA fragmentation [79].	E-TMS showed strong growth inhibition, particularly in COLO 205 cells. Apoptosis was associated with early ROS production followed by mitochondrial apoptotic signaling [79].

Abbreviations: PI3K, phosphoinositide 3-kinase; Akt, protein kinase B; MAPK, mitogen-activated protein kinase; JNK, c-Jun N-terminal kinase; NF-κB, nuclear factor kappa B; AP-1, activator protein-1; MMP-2, matrix metalloproteinase-2; ROS, reactive oxygen species; YAP1, Yes-associated protein 1; IGF2BP2, insulin-like growth factor 2 mRNA-binding protein 2; m6A, N6-methyladenosine; IC_50_, half-maximal inhibitory concentration. Concentrations refer to those reported in the original studies.

## Data Availability

No new data were created or analyzed in this study. Data sharing is not applicable to this article.

## Abbreviations

The following abbreviations are used in this manuscript:

AD	Alzheimer’s disease
ADAS-cog	Alzheimer’s Disease Assessment Scale–Cognitive Subscale
AE	Adverse event
BBB	Blood–brain barrier
CDR-SB	Clinical Dementia Rating–Sum of Boxes
CNS	Central nervous system
CSCs	Cancer stem cells
DCLK1	Doublecortin-like kinase 1
EGFR	Epidermal growth factor receptor
EMT	Epithelial–mesenchymal transition
FDA	Food and Drug Administration
HCV	Hepatitis C virus
IC_50_	Half-maximal inhibitory concentration
IUPAC	International Union of Pure and Applied Chemistry
JAK	Janus kinase
JNK	c-Jun N-terminal kinase
MAPK	Mitogen-activated protein kinase
MMSE	Mini-Mental State Examination
MMP	Matrix metalloproteinase
mTOR	Mechanistic target of rapamycin
NF-κB	Nuclear factor kappa B
NSCLC	Non-small cell lung cancer
ORR	Objective response rate
PI3K	Phosphoinositide 3-kinase
PRISMA	Preferred Reporting Items for Systematic Reviews and Meta-Analyses
ROS	Reactive oxygen species
RSV	Resveratrol
SAMDC	S-adenosylmethionine decarboxylase
SRT501	Micronized resveratrol formulation
STAT	Signal transducer and activator of transcription
T2DM	Type 2 diabetes mellitus
UGT	Uridine diphosphate-glucuronosyltransferase
SULT	Sulfotransferase
VEGFR2	Vascular endothelial growth factor receptor 2
Wnt	Wingless-related integration site
YAP1	Yes-associated protein 1
